# Current status of clinical trials for phage therapy

**DOI:** 10.1099/jmm.0.001895

**Published:** 2024-09-25

**Authors:** Chidiebere F. Uchechukwu, Adedayo Shonekan

**Affiliations:** 1 Warwick Medical School, University of Warwick, Coventry, UK; 2 University of Birmingham, Birmingham, UK

**Keywords:** antimicrobial resistance, bacteriophage therapy, clinical trials, infections, phage engineering

## Abstract

Recently, bacteriophages have been considered alternatives to antibacterial treatments. Infectious diseases continue to plague the world because bacteria can adapt and develop defence mechanisms against antibiotics. The growing incidence of antibiotic-resistant bacterial infections necessitated the development of new techniques for treating bacterial infections worldwide. Clinical trials have shown efficiency against antibiotic-resistant bacteria. However, scientists in future clinical trials should scrutinize phage resistance implications, assess combination strategies with antimicrobial agents and address challenges in phage therapy delivery for effective implementation.

## Introduction

Antimicrobial resistance (AMR) is a natural and ever-increasing threat to public health in the 21st century. According to the United Kingdom’s (UK) Review on AMR in 2016, an estimated 7×10^5^ people die each year globally from resistant infections, and a death toll of 10 million is projected by 2050 (https://amr-review.org/sites/default/files/160525_Final%20paper_with%20cover.pdf) [1]. Though this projection has been highly debated, the fact remains that the financial cost and impact on the lives lost by AMR grow and continue to grow steadily. Of particular concern is the presence of multidrug-resistant organisms, specifically those within the *Enterococcus faecium*, *Staphylococcus aureus*, *Klebsiella pneumoniae*, *Acinetobacter baumannii*, *Pseudomonas aeruginosa* and *Enterobacter* species family of pathogens.

Although strategies have been implemented to monitor, address and reduce the impact of this burden, the primary solution should be to find novel approaches to treat microbial infections. Conventional methods are subject to the ‘arms race’ between researchers and microbial evolution – as new drugs are tried and manufactured, microbes evolve to evade these targets. This creates the bottleneck and pressure associated with finding novel targets and keeping up with the evolutionary changes of microbes. In addition, conventional antimicrobial therapies tend to have unwanted side effects affecting the overall quality of life of the user (i.e. changes to commensal bacterial populations in broad-spectrum antibiotics). Recently, bacteriophages have been considered as alternatives to antibacterial treatments [2]. Phage therapy offers a viable and potentially safer alternative antimicrobial approach [3], boasting rapid and accessible discovery and more robust resistance to antimicrobial mutations and responses of the host bacteria.

### Phage therapy: basic mechanics

As mentioned earlier, phage therapy is an alternative antimicrobial approach. When introduced into the human body, they can act against target bacteria through several mechanisms despite the body’s immune defences. Here is an explanation of how bacteriophages can thrive and function in the human body: host specificity, rapid replication and action and localization at the site of bacterial infection. Unlike conventional/chemical antimicrobials, phage therapy uses native predators of bacteria to combat their disease burden on humans. Phages (bacteria-specific viruses) are abundant and naturally occurring. Like other viruses, phages cannot reproduce independently of their host species. After binding to the bacterial cell surface, phages will hijack the host mechanisms to either reproduce vertically (temperate phages), produce many daughter phages before lysing the cell (lytic phages) and releasing new phages that can propagate the cycle or integrate themselves into the host genome and remain in a latent phase until environmental stressors trigger the transition into the lytic phase [4]. Essentially, a temperate phage can select between two life cycles: the lytic cycle and the lysogenic cycle. The environmental factors determine whether a temperate phage undergoes the lytic or lysogenic cycle. When a temperate phage’s DNA is integrated into the host genome and stays inactive, it is called a lysogenic phage. Lytic phages are the preferred choice for therapeutic applications, as the other types of phages do not immediately address bacterial infection, and evidence suggests that temperate phages may confer increased resistance and virulence features to bacteria over time [5]. Due to the increasing resistance of the following strains to antibiotics and the need for alternative (and practical) treatment, most phage interventions in research and trial stages target strains of *P. aeruginosa*, *S. aureus* and, more recently, *Escherichia coli* [6,8].

### Clinical advantages of phage therapy over traditional approaches to microbial infections and current limitations

The current literature underscores several advantages of phage therapy over antibiotics. Research indicates that bacterial load decreases after phage treatment but remains constant after antibiotic treatment [9]. Phages improve cost, safety and long-term efficacy [10]. However, phages are also susceptible to recognition by the host immune system, leading to their clearance from circulation and significant reductions in available dosage [10]. One of the major encumbrances to the effectiveness of phage therapy is the presence of pathogenic bacteria that now show resistance to phages [11,12]. Several research studies have looked at the issue of phage resistance in bacteria, indicating that phage-resistant mutants are familiar and easily evade the bactericidal activity of phages [13,14]. The pharmacokinetics of bacteriophage therapy are far more complicated than those of other therapeutic methods.

However, despite the clear advantages offered by phage therapy as a solution to the ongoing issue of AMR, the practice has been slow to the uptake clinically in most of the Western world, with most phage applications being seen in Soviet countries such as Georgia [15]. These hurdles and considerations hinder its application because several critical parameters in phage therapy must be met, including phage adsorption rate, replication cycles, latency period, optimal phage dosing and administration route, which are obscured due to the elimination by some natural barriers (immune system), inter-phage variables, inter-individual differences and differential access to infection sites [16,17].

The slow uptake of phage therapy contrasts with the substantial emerging volume of data, underscoring its effectiveness. A recent systematic review encompassing clinical data revealed that out of 1904 patients who received phage treatment, over 79% exhibited clinical improvement, and successfully achieved bacterial eradication [18]. These findings carry particular significance, as most of these patients were contending with infections that had proven resistant to antibiotics yet were ultimately resolved through phage therapy [19,20].

This review aims to critically appraise the clinical applications of phage therapy. To do so, relevant clinical trials will be highlighted and discussed. Numerous case studies demonstrating the successful use of phage therapy are also available in the literature, and a range of these will also be presented. These case studies will include events where phage therapy has demonstrated itself as a viable option for niche applications, such as prosthetic devices and chronic infections. Due to the increasing interest in exploring phage therapy, many literature reviews exploring earlier trials and *in vitro* and *in vivo* studies in animal models are available [18,21,23]. Thus, in the interest of brevity, this report will limit the scope of this report to the last 5 years and only completed clinical trials (not those in recruitment or withdrawn due to funding without published results) will be highlighted.

## Clinical trials of phage therapies

**Fig. 1.**
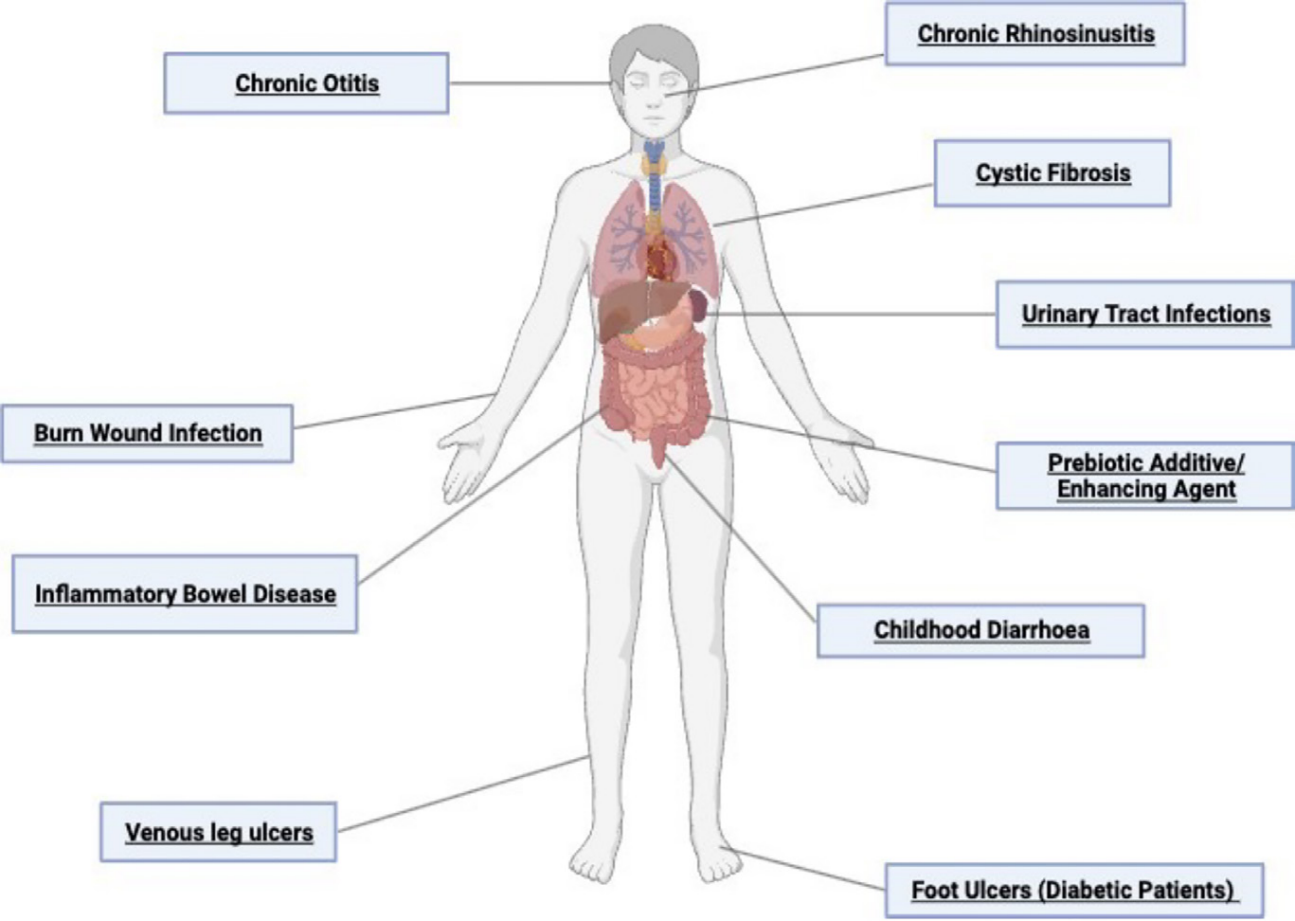
Clinical trials have employed phage therapy for various ailments in the relevant organ systems. Several clinical trials have used phage therapy as an intervention in the last 5 years. The indications for the clinical trials highlighted in this review span across ailments throughout the body, as highlighted in the figure. Created with BioRender.com.

The indications for phage therapy included chronic otitis and rhinosinusitis, cystic fibrosis, burn wound infections and others, as detailed in Fig. 1. At least 50% of the investigated clinical trials targeted *P. aeruginosa* infections, 29% targeted *E. coli* and the remaining trials focused on a mixture of other pathogens, including *Staphylococcus* spp., *Streptococcus* spp. and *Enterococcus* spp. Around 50% of the clinical trials investigated occurred in the United States of America, with the remaining in the UK, Georgia, France, Belgium, Israel or Australia.

All trials were in phase 1/2, with endpoints primarily including the safety and tolerability of the phage, number and severity of treatment-emergent adverse events and overall effectiveness of the therapy by various clinical/microbiological improvement measurements. All trials excluded pregnant patients, and participants of childbearing capacity were required to take effective forms of birth control for the duration and after the trial. No treatment-emergent severe adverse events were observed in any trials. Notable among these were three main trials: the SNIPR001 trial, the crPhage trial and the PreforPro trial.

The milestone crPhage trial (NCT04191148) was the world’s first clinical trial for a recombinant phage therapy [24]. Combining bacteriophage therapy with clustered regularly interspaced short palindromic repeats-associated protein 3 (CRISPR-Cas3) constructs targeting the pathogen’s genome to create the drug LBP-EC01, *E. coli* is targeted genetically via two efficient approaches. This dualistic approach was significantly more effective at killing *E. coli* than phage therapy alone *in vitro* and in small animal models. Around 1.5×10^10^ to 3.0×10^10^ p.f.u. per vial was dosed by intraurethral administration. Mixed results were observed in the trial, although, by day 2 of the treatment, 52.4% of participants in the treatment group compared to 28.6%, and by the end of the treatment, both placebo and treatment conditions had the same number of patients with improved cultures. Additional information on the trial can be found at https://clinicaltrials.gov/ct2/show/NCT04191148.

The PreforPro trial (NCT04511221) is one of the first clinical trials to explore the use of bacteriophages in healthy individuals to improve the efficacy of probiotic supplements [25]. The 93 participants with mild gastrointestinal (GI) symptoms (and otherwise healthy) were split into three groups and given either 4-week supply of 15 mg capsules containing probiotic and phage (1×10^9^ c.f.u. *Bifidobacterium animals* subsp. *lactis* and 1×10^6^ p.f.u. of commercial phage preparation PreforPro), 15 mg capsules containing filler material or 15 mg capsules containing probiotic only. Compared to comparators, the improvement of GI inflammation and colon pain in the experimental group was observed. By combining phages against *E. coli* strains with daily probiotic treatments over 4 weeks, this trial highlighted the utility of phages as a supplement.

The SNIPR001 trial (NCT05277350) uniquely engineered bacteriophages to deliver CRISPR/Cas targeting the essential components of *E. coli* strains found in patients with urinary tract infections [26]. This study demonstrated the safety and tolerability of using both the lytic activity of phages and the gene editing capacity of CRISPR/Cas9 systems to tackle persistent infection. Table 1 shows all details of the investigated studies and clinical trials completed between 2018 and 2023 using phage therapy to address many ailments.

**Table 1.** Clinical trials completed between 2018 and 2023 using phage therapy to address a myriad of ailments

Study, location(s) and clinical trial no. (if appropriate)	Study type	Sample size	Targeted pathogen(s)	Ailment	Recruitment criteria	Objective and primary outcome measure(s)	Phage name, cocktail or individualized	Mode, dosage, and duration of treatment	Placebo/comparator treatment	Key results	Reference no.
USA, 2022 (NCT05277350)	Double blind, dose escalation, randomized, dose escalation, phase 1	*N*=36	*E. coli*	n/a	Healthy (no clinically relevant abnormalities or disease diagnoses), 18–65 years old, *E. coli* present in faecal samples, normal defecation pattern	Safety and tolerability of phage; measured by incidence and severity of AEs and medically attended adverse events from the first administration of study drug and up until day 35 of the study	SNIPR001, cocktail	Twice daily for 7 days	Undefined	Oral dosing over 7 days was well tolerated with only mild-to-moderate side effects and no withdrawals	[10]
USA, 2020 (NCT04511221)	Randomized, double blinded, placebo-controlled, parallel arm, triple blind	*N*=93	*E. coli*	Mild GI symptoms	Otherwise, healthy, 18–65 years old, BMI 20–34.9	GI symptoms assessed via questionnaire; bowel function was assessed by a record of all stools (Bristol Stool Scale); microbiota analysis was analysed via 16S sequencing; phage plating was used to assess phage activity	PreforPro, cocktail	15 mg capsule containing 1×10^9^ c.f.u. *B. animals* subsp. *lactis* BL04 and 1×10^6^ p.f.u of PreforPro (commercial phage preparation) with rice maltodextrin and medium chain coconut triglycerides as a filler material; one tablet taken orally each day for 4 weeks	Placebo: 15 mg capsule containing rice maltodextrin and medium-chain coconut triglycerides; active comparator: 15 mg capsule containing 1×10^9^ c.f.u. *B. animals* subsp. *lactis* BL04 with rice maltodextrin and medium-chain coconut triglycerides as a filler material	Improvement of GI inflammation and colon pain in the experimental group compared to comparators	[11]
USA, 2022 (NCT04596319)	Multi-centre, double blind, randomized, placebo- controlled study, phase 1/2	*N*=29	*P. aeruginosa*	Cystic fibrosis and chronic pulmonary *P. aeruginosa* infection	Documented diagnosis of cystic fibrosis, 18 years of age or older, forced expiratory volume greater than 60% for single-dose treatment and 40% for multiple-dose treatment	Incidence of treatment-emergent adverse events of single and multiple doses of phage therapy administered by inhalation	AP-PA02, cocktail	Unspecified dosage	Inactive isotonic solution	Patients treated with AP-PA02 showed a trend towards a lower bacterial load after 10 days compared with patients given a placebo; AP-PA02 was well tolerated, with a similar profile of adverse events between the placebo and intervention arm	[12]
USA, 2021 (NCT04191148)	Multi-centre, randomized, parallel assignment, double blind, phase 1	*N*=36	*E. coli*	Patients with indwelling urinary catheters or requiring intermittent catheterization and/or patients with asymptomatic bacteriuria caused by *E. coli*	18 years or older, male or female, with lower urinary tract infections caused by *E.coli* but otherwise healthy; experience with urinary catheterization desirable	Safety and tolerability of LBP- EC01: number of participants with treatment-related adverse events as assessed by DAIDS version 2.1	LBP-EC01, cocktail	Approximately 1.5×10^10^ to 3.0×10^10^ p.f.u. per vial dosed twice daily by intraurethral administration for 28 days	Lactated Ringer’s solution	No treatment-related adverse events (assessed by DAIDS v 2.1); by day 2, 11/21 (52.4%) analysed participants experienced a reduction in urinary *E. coli* burden (defined by at least 1 log c.f.u. reduction from baseline) compared to 2/7 (28.6%) in the placebo group; by the end of the treatment (day 28), 9/21 (42.9%) had experienced a reduction in urinary *E. coli* burden (defined by at least 1 log c.f.u. reduction from baseline), compared to 3/7 (42.9%) in the placebo group	[13]
Israel, 2022 (NCT04803708)	Double blind and randomized study, phase 1/2	*N*=20	*P. aeruginosa*, *S. aureus* and/or *A. baumannii*	Treatment of non-infected and infected diabetic foot ulcers	18 years or older; male or female; T1D or T2D with glycated haemoglobin; suitable physical and mental health, medically stable; infected or non-infected diabetic foot ulcers	Incidence and severity of treatment-emergent solicited local and systemic AEs and relationship to IP from first administration until 1 week after the end of the treatment	TP-102, cocktail	1 ml intraperitoneal solution applied topically at 1×10^9^ p.f.u. ml^−1^; non-infected ulcers: applied thrice daily every other day for a week; infected ulcers with grade 2 or 3 infection: applied thrice daily every other day for up to 4 weeks	Unspecified	No treatment-related SAEs were observed in either arm; in the infected ulcer group, 50% of the subjects showed significant reduction or eradication from the baseline in microbiologic data via culture (c.f.u.) at d3, d8, d15, d22, d26 and end of the study; the median time to significant reduction/eradication of target bacteria via culture was 22 days for the infected ulcer group; about 74.6% of patients in the treatment arm showed closure of the wound compared to 34.2% in the placebo arm	[14]
USA, 2021 (NCT04737876)	Randomized, single blind, placebo controlled, phase 1	*N*=18	*K. pneumoniae*	Inflammatory bowel disease/primary sclerosing cholangitis	Healthy adults (18–65 years of age)	Safety and tolerability of phage preparation	BX002-A, cocktail	2.8×10^10^ total p.f.u. per dose; administered twice daily for 3 days; participants also received oral esomeprazole once daily for the duration of the treatment to optimize gut pH conditions for phage survival	Unspecified	No serious treatment-emergent adverse events recorded	[15]
Australia, 2019 (ACTRN12616000002 482)	Single-centre, first-in-human, open-label clinical trial of multiple ascending doses	*N*=9, three patients per cohort in three cohorts	*S. aureus*	Chronic rhinosinusitis	18–70 years old, recalcitrant CRS in whom surgical and medical treatment had failed and who had positive *S. aureus* cultures sensitive to intervention phage (AB-SA01)	Safety and tolerability of intranasal AB-SA01	AB-SA01, cocktail	Intranasal irrigation of phage cocktail AB-SA01; delivered twice daily in varying durations and concentrations depending on cohort; cohort 1: 3×10^8^ p.f.u. for 7 days; cohort 2 : 3×10^8^ p.f.u. for 14 days; cohort 3 :3×10^9^ p.f.u. for 7 days	n/a	Safe and well tolerated at all doses and durations; mild treatment-emergent adverse events observed, resolved by the end of the study; 2/9 of patients experienced eradication of infection	[16]
USA, 2020 (NCT04684641)	Prospective, randomized, placebo- controlled, double blinded, single-site study	*N*=8	*P. aeruginosa*	Cystic fibrosis subjects with chronic *P. aeruginosa* airway infections	18 years or older, male or female, cystic fibrosis diagnosis with *P. aeruginosa*-positive cultures, able to provide repeated induced sputum samples and use a nebulizer, clinically stable lung disease	Reduction in sputum bacterial culture (measured by c.f.u. per millilitre); safety and efficacy of phage intervention	YPT-01, cocktail	3 ml phage therapy, nebulized daily for 7 days; unknown dosage	Unspecified	Ongoing trial; initial data demonstrate well-tolerated treatment, with minimal and resolved treatment-emergent adverse side effects; the trend towards improvement in forced expiratory volume in intervention arm participants compared to placebo arm participants	[17]

AEAdverse eventBMIBody mass indexCRSChronic rhinosinusitis DAIDSDivision of AIDS GIGastrointestinal IPIntraperitonealSAESerious Adverse EventT1DType 1 diabetesT2DType 2 diabetes

## Successful case studies and series of phage therapy

The indications for phage therapy in the explored case studies included foot ulcers, cystic fibrosis, chronic rhinosinusitis, multidrug resistant infections, opportunistic infections after immunosuppression and chronic and recurring infections of prosthetic joints with and without the presence of biofilms [27,32]. About 42% of case studies explored *P. aeruginosa* infections, with the remaining case studies including other well-known pathogens such as multiple drug-resistant *S. aureus*, *E. coli, Klebsiella pneumoniae *and *Mycobacterium abscessus* [57-62]*.*

Modes of phage administration were typically suited to the primary ailment (i.e. pulmonary infections were given phage treatment primarily via a nebulizer, and prosthetic limb infections had infection sites washed and incubated with phage in solution). However, intravenous (IV) and topical modes of administration were also employed. All cases reported no adverse events, and complete resolution of infection without relapse was observed despite the inability of prior treatments to achieve this outcome. About two of the nine case studies used phage therapy in combination with antibiotic treatment during and after the course of phage therapy. The duration of follow-up in these studies ranged from 20 days to 6 months. All details of the investigated studies are shown in Table 2.

**Table 2.** Successful case studies completed between 2018 and 2023 employing the use of phage therapy to address a myriad of ailments

Study and location(s)	Patient details	Targeted pathogen(s)	Ailment	Phage name, cocktail or individualized	Mode and duration of treatment	Key results	Reference no.
Fish *et al*., 2018, USA	*N*=6	*S. aureus*	Foot ulcers	Sb-1, cocktail	Undisclosed concentration; bacteriophage preparation was dripped into the wound cavity, which was then packed with plain packing gauze soaked with bacteriophage preparation (0.1–0.5 cc), covered with specialized gauze to prevent solution wicking; the dressing with packing was kept in place for 48 h, and then, the patient performed dressing changes with standard moist dressings for the balance of the week; bacteriophage reapplied during weekly meetings	To date, all patients treated with bacteriophage have successfully healed where conventional therapy had previously failed; no adverse effects, tissue breakdown or recurrence of infection was seen, and closure was smooth and continuous after the initiation of bacteriophage therapy	[18]
Dedrick *et al*., 2019, UK	15-year-old male	*M. abscessus* (designated strain GD01)	Cystic fibrosis, chronic *M. abscessus* infection prior to and following bilateral lung transplantation	Anti-*M. abscessus* phage, cocktail	Cocktail diluted in PBS to a concentration of 10^9^ p.f.u. ml^−1^, administered IV every 12 h for at least 32 weeks; skin lesions also received a topical dose of the cocktail at 10^9^ p.f.u. per 7 ml	Phage treatment is well tolerated throughout treatment duration, improved lung and liver function, healing of skin lesions and transplant incision sites; no evidence of phage neutralization in sera and weak antibody responses to phages was observed	[21]
Doub *et al*., 2020, USA	72-year-old male with morbid obesity (BMI 41) and hyperlipidaemia	MRSA	Chronic infection of prosthetic joints; recurring infection despite repeated debridement, antibiotics and implant retention (DAIR) and antibiotics	SaGR51φ1, single phage	Two doses of IA bacteriophage (5.4×10^9^ p.f.u.) in 10 ml of normal saline (NS) and were started on IV daptomycin 1000 mg daily; daily IV bacteriophage (2.7×10^9^ p.f.u. in 50 ml of NS) was also administered; therapy was stopped after 6 weeks due to significant transaminitis	Successful treatment is confirmed by negative cultures of the joint fluid and surrounding tissues; the patient's symptoms also improved, and he was able to walk without pain and return to work	[22]
Cano *et al*., 2021, USA	62-year-old diabetic man with a history of right total knee arthroplasty	*K. pneumoniae*	Multiple episodes of prosthetic knee infection with biofilm formation despite numerous surgeries and prolonged courses of antibiotics, with progressive clinical worsening and development of severe allergies to antibiotics	KpJH46Φ2, single phage	40-week daily administered IV doses (6.3×10^10^ phages in 50 ml) targeting the specific bacterial isolate, alongside continued minocycline for 8 weeks	Phage therapy resulted in the resolution of local symptoms and signs of infection and recovery of function; the patient did not experience treatment-related adverse effects and remained asymptomatic 34 weeks after completing treatment while still receiving minocycline	[23]
Onsea *et al*., 2019, Belgium	*N*=4	*Enterococcus faecalis*, *S. aureus*, *P. aeruginosa*	Severe musculoskeletal infections (osteomyelitis)	Commercially available Pyo bacteriophage cocktail and BFC1 phage cocktail	Phages diluted in 0.9% saline to a titre of 10^7^ p.f.u. ml^−1^; 10–40 ml phage solution was used to rinse the wounds, followed by 10 min of contact time and drainage; the gentamicin-impregnated sponge was then soaked in phage solution and placed in contact with the wound before closure; the treatment was employed thrice daily for 7–10 days; all patients received concomitant antibiotic therapy	No severe side effects were noted; after a single course of phage therapy with concomitant antibiotics, no recurrence of infection with the targeted strains occurred, with follow-up periods ranging from 8 to 16 months	[24]
Cesta *et al*., 2023, Italy	62-year-old female	*P. aeruginosa* Pa_AR1, a strong biofilm producer	Chronic right hip prosthesis infection	Pa53, personalized cocktail	Self-administered (10^3^ p.f.u. ml^−1^) with meropenem	No further adverse reactions in the next administrations were reported after the reduction in phage volume; no pain or local inflammation present in the hip, and no signs of infection relapse with the patient in good clinical condition	[25]
Ferry *et al*., 2021, France	88-year-old man	Relapsing *P. aeruginosa*	Prosthetic knee infection	Unnamed, personalized mixture of two unnamed phage cocktails	Final dilution 1×10^9^ p.f.u. ml^−1^; 30 cc administered via injection through the arthroscope to the site of infection (PhagoDAIR approach), alongside IV ceftazidime and then oral ciprofloxacin as suppressive antimicrobial therapy	Patient rapidly improved, with the disappearance of pain in the left knee; during the follow-up of 1 year, the local status of the knee was normal, and its motion and walking were unpainful	[26]
Tkhilaishvili *et al*., 2019, Germany	80-year-old woman with metabolic syndrome	Two morphologically distinct MDR *P. aeruginosa* isolates	Periprosthetic joint infection	Unnamed, single phage	Single 100 ml loading dose applied locally during surgery; 5 ml of 10 ^8^ p.f.u. ml^−1^ phage solution applied every 8 h via local delivery systems for 5 days; used alongside antibiotics throughout the treatment	Combined phage-antibiotic therapy was well tolerated, resulting in microbiological eradication, whereas repeated previous courses of antibiotics and surgical treatment failed	[33]
Doub *et al*., 2021, USA	79-year-old female	Recalcitrant *S. epidermidis*	Prosthetic knee infection	PM448, ε^2^ phage cocktail	2×10^10^ p.f.u. in 10 ml saline injected to space around the prosthesis; treatment in combination with DAIR and IV antibiotics; the initial plan included continuous IV phage dosing for 4 days but was declined by the patient due to the increase in aspartate aminotransferase and alanine aminotransferase	Treatment was well tolerated with no significant adverse reactions; 5 months after DAIR and adjuvant bacteriophage therapy, the patient has a full range of motion of her knee and no clinical signs of PJI recurrence	[34]
Ferry *et al*., 2020, France	*N*=3	*S. aureus*	Relapsing prosthetic knee infections	Unnamed, three phage cocktails	1×10^9^ p.f.u. ml^−1^, which was administered by the surgeon directly into the joint, after the DAIR procedure and joint closure (PhagoDAIR procedure); continued use of antibiotics throughout and after the treatment	New DAIR required for two patients, but no additional phage applied; follow-ups at 7, 11 and 30 months displayed improvement in knee function and symptoms for all patients; at the end of the follow-up period, total disappearance of signs of infection was noticed in two out of the three patients; the remaining patient had a persistent fistula containing synovial fluid	[35]

BMIBody mass indexDAIRDebridement, antibiotics and implant retentionIAIntra-arterialMDRMultidrug resistanceMRSAMethicillin-resistant Staphylococcus aureus

Three case studies of note provide critical insights into the functionality of phage therapies and best practices for their use in niche applications. In Italy, Cesta *et al*. demonstrated that phage therapy combats biofilms, albeit a higher titre is required [33]. However, it is essential to note that after 24 h of incubation, phage resistance appears to have developed. *In vitro* work also demonstrated the potential for phage resistance to develop in long-term (24 h) exposure to phage cocktails. Thus, it was concluded that phage alone could not eradicate long-term biofilm. However, combining phage and meropenem was more effective in combatting biofilm than either meropenem alone or phage therapy alone.

Also of note is the case series by Ferry *et al*. that introduced the ‘PhagoDAIR’ approach for treating persistent prosthetic joint infections [34]. In this approach, a phage solution is applied to the site of infection immediately following debridement, antibiotics and implant retention (DAIR) intervention and joint closure. Two out of three patients experienced complete resolution of the infection and associated symptoms, and one patient had partial resolution with a fistula still present. This approach is being trialled in France on a larger clinical trial, and the initial results are expected to be available by 2024.

Another case series in Belgium put forth a ‘multidisciplinary phage task force’ and standardized treatment pathway for phage treatment of severe musculoskeletal infections based on the treatment of four patients [35]. Through the collaboration between clinicians and phage scientists, phage cocktails were applied intraoperatively, and, using a drainage system, the cocktails were applied thrice daily to the site of infection for 7–10 days. All patients responded well to the treatment and experienced a complete resolution of infection without recurrence.

## Discussions

Antibiotic resistance is a growing cause for concern worldwide, necessitating novel and alternative therapeutic approaches to combat the causative pathogens. However, the traditional drug design requires both considerable time and financial resources, and there are periods of stagnation before new generations of drugs can be designed. Phages are naturally occurring, abundant entities that combat specific strains of bacteria and have emerged as a viable alternative/adjuvant to traditional antibiotic therapies. This review has explored clinical trials, case studies and series, as well as compassionate use cases that highlight the detailed and practical work employing phage therapies and the importance of continued work and an established protocol for their administration in a clinical setting. In turn, this literature has highlighted considerations from bench to bedside that must be taken to move forward with the routine use of phage in clinical settings. These considerations will be detailed briefly below.

### Dosage

The effective dosage of phages in clinical applications can be a complex subject. Various factors, including the specific pathogen, patient characteristics and the route of administration, play pivotal roles in determining the correct dosage. Additionally, the diverse approaches to phage therapy and inconsistent reporting practices complicate the establishment of standardized dosing guidelines.

It has previously been observed that a lower phage dose could lead to the selection of phage-resistant bacteria, while a higher dose achieved a more favourable outcome [36]. These findings are supported by Cesta *et al*., who demonstrated that higher phage litres were essential in treating infections with biofilm formation [33]. However, this treatment used a different phage preparation and was administered in combination with meropenem, so it is only possible to partially attribute the results to the high titre alone. The minimum effective dosage observed in this review was a 10^7^ p.f.u. ml^−1^ titre used to treat four patients with severe musculoskeletal *Enterococcus faecalis*, *S. aureus* and *P. aeruginosa* infections [35].

To navigate this complexity, it is crucial to generate more research that considers factors such as the infection site’s role, biofilms’ presence and the pathogen’s nature in active dosage. Combining this information with an understanding of phage pharmacokinetics can help guide the establishment of appropriate dosing regimens for phage therapy.

### Preparation methods: purification, susceptibility testing and storage

Preparation methods play a significant role in the safety and efficacy of phage therapy and the received dosage. The importance of quality phage preparation methods and reporting of those methods is highlighted in the recent PhagoBurn study, where a fourfold reduction in the expected dosage of PP1131 during the trial impacted its therapeutic efficacy [37]. The causative details of the instability were not made available. This emphasizes the importance of monitoring the stability of the final product (phage cocktail).

There are also inconsistencies in the literature’s level of detail regarding phage preparation. Most studies reported phage concentration (p.f.u. per millilitre) at a minimum. Still, the majority did not provide phage or bacterial host genotype information, endotoxin contamination levels of final phage preparation, phage filtration processes or results of susceptibility testing. These are all essential data for safety and efficacy reasons and should, therefore, be incorporated into good practice for phage use. Onsea *et al*. is an excellent example of delivering the above and may be used for reference [35].

### Cocktails vs. individualized

The decision between employing individualized phage therapies or cocktails in clinical applications is multifaceted, with each approach carrying distinct advantages and limitations that must be carefully considered. Individualized phage therapy offers a precise treatment strategy, tailoring phage selection to the patient’s infecting pathogen. This approach reduces the risk of phage-resistant mutants but can be time-consuming and relies on available well-characterized phages.

Conversely, phage cocktails provide a broader spectrum of activity against various pathogens, making them suitable when the infecting strain is unknown or multiple pathogens are involved.

The effectiveness of a phage cocktail was evaluated by treating mice that had suffered after being infected with *K. pneumoniae* at a fatal dose. The phages were isolated from both wild-type bacteria and phage-resistant strains. After inoculation with bacteria, a single intraperitoneal dosage 1 h later produced a complete recovery [38]. This also makes them suitable for broad use in patients with infections from the same strain, thus minimizing the burden of designing patient-specific phages. However, challenges include optimizing dosages, preventing phage interactions and the emergence of resistance.

It is worth noting that all clinical trials reviewed in this study and those currently recruiting apply phage cocktail therapies. Ribeiro *et al*. suggested that integrating strategies and techniques to comprehensively assess the host range and lytic activity of bacteriophages under different conditions can demonstrate more accurately the antibacterial potential of phage cocktails [39]. This suggests that, in practice, the cocktail approach is more useful in a more comprehensive clinical setting. Conversely, the case studies either employ personalized cocktails or targeted individual therapies. Both methods have been reported to be well tolerated and effective [40,41]. Thus, clinicians and researchers should evaluate factors like phage availability, treatment urgency and resistance risk to choose the most suitable approach for each case. In terms of feasibility for regulatory and commercialization purposes, however, streamlined cocktail approaches appear to confer a more considerable advantage [40,42]. Individualized therapies can then be applied on a case-by-case basis after unsuccessful cocktails.

### Combination therapy vs. individual use

Phage therapy, when combined with antibiotics, can reduce the emergence of phage-resistant subpopulations. Administering phages alongside other treatments, particularly antibiotics, has created a synergistic effect in some studies, particularly when phages precede antibiotics [43,44]. However, not all studies confirm this synergy between phages and antibiotics [45], and some suggest that the choice of antibiotics plays a critical role in the effectiveness of combination therapy [46].

Due to the relatively novel use of phage therapy on a larger scale, clinical trials have focused mainly on using phages without antibiotics. In case studies, combination therapies appear to be more frequently used, where there is treatment for chronic joint infections with the presence of biofilms, with success. However, as observed with Doub *et al*.’s patient, the antibiotic portion must sometimes be halted due to the emergence of transaminitis [47]. More studies assessing the benefits of combination vs. individual therapy should be done to explore the nuances of this approach further. Following the success of the SWARM-Pa trial [48], a follow-up Tailwind study comparing combination antibiotic-phage therapy against phage therapy alone indicated an awareness of the need for more comparative data in this area [49]. Unsolved puzzles remain regarding the long-term effects of such combination therapy. The concern revolves around the likelihood of bacterial mutation and subsequent resistance to phage-antibiotic therapy. The indication of this concern is that when selecting a therapeutic combination of phage and antibiotics, the ability of the combination to cause resistance to the phage-antibiotic therapy is dose dependent and should be considered [50].

### Safety and toxicity

In the context of clinical applications of phage therapy, our review underscores the importance of addressing safety and toxicity concerns. Cesta *et al*. observed an onset of high-grade fever following the initial phage administration at high litres that reduced after lowering the titre [33]. This may be attributed to the rapid accumulation of pyrogenic toxins (perhaps due to residual endotoxins from the formulation). Similar reports of fever in phage therapy have been observed and attributed to the release of endotoxins during the lytic phase of phage replication in host bacteria [51]. This emphasizes the necessity of rigorous endotoxin assessment and reporting during phage preparation to ensure patient safety.

Standardized safety assessments are indispensable in animal and human phage therapy studies. Few clinical trials discussed in this review identified key safety parameters such as the ED50, LD50 and therapeutic index. However, this may be partly due to the ongoing ambiguity surrounding phage dose studies, which are discussed later. Moreover, data on the effects of phage therapy on pregnancy, growth and development were notably scarce, primarily confined to rodent models, and participants of all clinical studies of childbearing capacity were asked to go on birth control for the duration of the study.

A more comprehensive understanding of safety requires reporting adverse events, laboratory results, vital signs and physical examinations in clinical evaluations. Moreover, given the phages’ immunogenic nature, it is imperative to assess their impact on immune responses, including the incidence and consequences of neutralizing antibodies and potential adverse events related to their formation and reactions. Incorporating these safety assessments into both animal and human studies is not only advisable but also essential for advancing the field of phage therapy.

### Mode of application

Several studies employed the intravenous (IV) phage application [52,53]. However, this method has its limitations. IV application, while effective, restricts the potential for patients to self-administer phage treatments at home. Additionally, IV application may lead to reduced delivery of active phages to the intended site, complicating the determination of the working dosage and effective dose. In contrast, the direct application of a phage solution to the site of infection, primarily utilized in cases of prosthetic limb infections after DAIR procedures, offers the advantage of precise phage delivery directly to the infection site [54]. This approach may allow a more accurate approximation of the delivered phage concentration. In instances where biofilm formation is present, this method can be further optimized by pre-rinsing the site with sodium bicarbonate to re-establish a suitable pH environment within the acidic biofilm-associated infection, as detailed by Ferry *et al*. [55]. However, this technique requires patient immobilization for a specified duration to maintain effective contact between the solution and the infection site.

Another alternative involves using phage-impregnated bandages, as employed by the successful case series reported by Fish *et al*. [56]. However, there needs to be more reported data on the release of phages from these bandages and their reflection of the actual administered dose. Phages may become trapped in the gauze, hindering their release and subsequent bacteriolytic action. Alternatively, spray devices could be employed to aerosolize liquid phage preparations directly onto the wound infection. Phage spray applications have primarily been used in food preservation for fruit, meat and cattle hides, and their adaptation for wound infection treatment holds potential. Phages have also been formulated in gels or creams and were to be used in a clinical trial in the UK, but the trial was halted due to funding shortages [57].

Finally, Cesta *et al*. highlight the potential for self-administration to be a successful mode of treatment [33]. Still, this approach on a larger scale may mean patients need clear therapeutic protocols and medical follow-up to manage their phage treatment.

### Future directions

*In vivo* panning studies for using phages in tissue regeneration [58] are being considered because phages do not parasitize humans. This is an emerging research area in phage engineering [58]. Vaccine production is another emerging use of phage technology. Recently, phage engineering has been employed to create vaccines. The primary technique for using phage to create vaccine designs is phage display [59,60]. A copy of the antigen’s genetic molecules is incorporated into the phage DNA for phage-engineered vaccines. Once inside the host cell, the genetic molecules of the antigens are expressed by the same process the host cell uses to make its proteins. The immune system detects antigen expression on the phage surface and mounts an immune reaction against the antigen [59]. A cell-free synthesis technology called cell-free transcription–translation has recently made significant advances in addition to phage engineering and synthesis technologies [61]. Using a helper phage-free transducing particle preparation as an antimicrobial agent will pave the way for developing new phage-based technologies with greater scope than lytic phage therapy [62]. Arming phages with heterologous effectors will pave the way for successful Urinary tract infection treatment and represents a versatile tool to enhance and adapt phage-based precision antimicrobials [63].

## Conclusion

This review underscores the growing interest, need and capacity for further research to establish phage therapy as a viable tool for bacterial pathogen treatment. Notably, many actively recruiting clinical trials and those with impending recruitment phases indicate the ongoing momentum in this field. These studies hold promise, particularly in cases where alternative treatments might have resulted in significant patient harm or diminished quality of life, such as total joint arthroplasty. From the patient’s perspective, a survey (although small scale) has revealed a patient’s willingness to try phage therapy if a doctor recommends it, with some suggesting they would even be likely to try it before antibiotic interventions [64].

Moreover, the global landscape of phage therapy is evolving rapidly. The increasing number of planned clinical trials and phage therapy units reflects the continued interest in integrating phage therapy into routine disease treatment. This marks an exciting and transformative period for the field of phage therapy.

## Abbreviations

AMR: antimicrobial resistance
DAIR: debridement, antibiotics and implant retention
GI: gastrointestinal
IV: intravenous
